# Evolution of Research on Global Soil Water Content in the Past 30 Years Based on ITGinsight Bibliometric Analysis

**DOI:** 10.3390/ijerph192315476

**Published:** 2022-11-22

**Authors:** Xifeng Zhang, Shuiming Liang, Jiaqi Lu, Xiaowei Cui

**Affiliations:** 1 College of Geography and Environment Science, Northwest Normal University, Lanzhou 730070, Gansu Province, China; 2 Key Laboratory of Resource Environment and Sustainable Development of Oasis, Lanzhou 730000, Gansu Province, China

**Keywords:** soil water content, bibliometric analysis, ITGinsight, network map analysis

## Abstract

Research on soil water content (SWC) has involved a wide range of disciplines and attracted constant attention. Current literature reviews primarily focus on a specific type of research on SWC and few systematic studies have been performed to fully evaluate the development and changes in hotspots of SWC research. In this study, a bibliometric analysis and visualization are used to understand the development of SWC research in countries of Europe, Asia, Oceania, and North America. The research data came from the Web of Science database and the time span was 1987–2021. Since 1987, the numbers of international SWC research papers have increased rapidly. The United States and China have the closest exchanges and most publications in the field of SWC. Keyword network maps indicated that early research on SWC was mostly in small-scale farmlands and woodlands, with diverse research hotspots including those focused on SWC stress, soil physical modeling, soil hydrothermal processes, and SWC measurement. Due to climate change, remote sensing technology development, and policies, research on SWC gradually focused on watershed, regional, and global scales, with research hotspots including those focused on evapotranspiration, land–air energy exchange, and remote sensing satellite inversion of SWC products. In addition, in recent years, the research of SWC and SMAP has attracted considerable attention worldwide. The United States has more influence in the SWC sector than China. Although the number of articles that have been published by European countries was small, the influence of those papers should not be underestimated.

## 1. Introduction

As a fundamental variable in mass transfer and energy exchange in groundwater–soil–plant–atmosphere interactions, the soil water content (SWC) has direct effects on plant growth, agricultural production, hydrological processes, soil erosion, land degradation, and climate change, and is listed as one of the 50 Essential Climate Variables by the Global Climate Observing System [1]. Thus, reviewing of the changes of SWC research hotpots around the world can provide a theoretical basis for implementing water use measures and managing land resources.

Although many articles have been published on SWC in recent years, there are few review articles. Raats et al. [2] reviewed the classical theory of Richards [3] and listed some developments extending beyond that theory, emphasizing its importance in soil physics. Daly and Porporato et al. [4] reviewed the manifold repercussions of SWC dynamics on vegetation response and biogeochemical cycles. Seneviratne et al. [5] specifically focused on SWC–precipitation feedback and possible modifications of feedback with climate variability and extreme events and emphasized the importance of quantification. Dobriyal et al. [6] reviewed the estimation method of SWC at the landscape level and concluded that measurements of time domain reflectometry (TDR) and ground penetrating radar (GPR) methods were instantaneously obtained and accurate. Owing to the high spatial and temporal variability of SWC [7], temporally and spatially continuous SWC datasets are challenging to develop and validate using ground-based measurements alone. Remote sensing has become an alternative approach to estimate SWC [8]. There are an increasing number of review studies on the relationship between SWC and remote sensing focus on applied hydrology [9], precision agriculture [10], and disaster prevention [11].

Traditional literature reviews primarily focus on a specific type of research on SWC and, therefore, fail to fully reflect the development and changes in hotspots of SWC research. Thus, it is necessary to conduct a quantitative analysis to summarize the research status on SWC in order to orient future research directions.

This study used a keyword professional search method to search the literature in the Web of Science (WOS) database. The study used the ITGinsight literature visualization analysis software that was developed by Professor Liu Yuqin and his team with its powerful literature cooperation analysis, co-occurrence analysis, and other functions [12]. The software scientifically, objectively, quantitatively, systematically, and intuitively sorts the development context and changes in research hotspots of SWC research in different periods and regions of the world and identifies existing problems and future directions SWC research.

## 2. Materials and Methods

### 2.1. Data Acquisition

Keywords outline the core content of literature and can represent the characteristics of research on soil water content. In this study, the advanced keyword retrieval in the WOS platform was used, and it selected literature in English from 1987 to 2021 as the research object. The “Author Keywords (AK)” query of the WOS was “AK = (“soil moisture” OR “soil water” OR “soil humidity” OR “soil water content”)”. The data were retrieved on 10 December 2021. “Paper” was selected as the literature type. The discipline category was the category with more than 100 articles. After screening, the literature that was unrelated to keywords was deleted, and as a result, 15,195 records were obtained.

### 2.2. Methods of Data Analysis

#### 2.2.1. Co-Occurrence Network Method

The software ITGinsight_V2.0_R64 (http://cn.itginsight.com/ (accessed on 10 December 2021.)) was used to analyze the country, institution, and keywords knowledge graph of the 15,195 articles. ITGinsight is an advanced scientific and technological text mining and visualization analysis tool that is used primarily for patents, papers, reports, newspapers, and other scientific technological text data mining and map establishment [12]. Compared with CiteSpace and Hitcite, ITGinsight has significant advantages because of its unique user glossary, evolution analysis, and diversified visualization performance. In this study, ITGinsight was used to analyze national co-authorship, institutions of co-authors, and keyword contribution network visualization.

The data were extracted and analyzed year by year, and other operations were set as the default settings to obtain the relationship graph of different periods. The clustering structure feature of the relationship atlas was reflected in nodes. Node color is used to indicate the number of articles with different keywords, the size of nodes indicates the number of publications, and the thickness of lines between nodes indicates the number of co-authorships, with larger lines indicating greater co-authorship, i.e., a higher level of cooperation. On the basis of characteristics of regional dispatch volume, this study analyzed the frequency and mutation types of each keyword in SWC research in the three major dispatch regions of Asia and Oceania, North America, and Europe one by one in three time periods (1987–1998, 1999–2010, 2011–2021). The results revealed the research history of SWC in different regions at different stages. 

#### 2.2.2. Keywords Temporal Trend Analysis

The trend analysis of keywords helps to better understand the time evolution of the research topic. In order to better present the data, we divided the 30-year data into two stages (1987−2014, 2015− 2021) in terms of the number of articles, and the number of articles in both stages was between 7000 and 8000. If the current (2015–2021) keyword appears frequently, but in the past (1987–2014) the frequency is low, the increasing frequency indicates that the keyword is more likely to be a trend. To extract and visualize keywords with rising trends, the normalized cumulative keyword frequency (*f*) was calculated based on the keyword frequency (*F*) and the number of papers (*N*) per year. The normalized frequency makes it possible to provide a fair comparison of topics across time periods, as the number of annual publications changes over time. Past (*F_P_*) or current (*F_R_*) normalized cumulative keyword frequencies were defined as the number of keyword-related papers per 1000 papers in the past or current period, respectively. To reflect trends, we use an indicator, the trend factor, calculated as the log of the ratio of *F* current to *F* past [13]:

(1) $$F_{R}=1000\times \frac{\sum_{2015}^{2021}f}{\sum_{2015}^{2021}N}$$

(2) $$F_{P}=1000\times \frac{\sum_{1987}^{2014}f}{\sum_{1987}^{2014}N}$$

(3) $$T=\log\frac{F_{R}}{F_{P}}$$

Therefore, *T* > 0 means that the frequency of keywords increases, that is, keywords are more likely to be on the rise, and vice versa. OrginPro 2022 visualizes the trends of the top keywords and further analyzes the rising keywords and their specific time trends. This method was used to determine the dynamic changes of the time span (1987–2014) and (2015–2021) worldwide to fully explore the hotspot changes in soil water research.

#### 2.2.3. Citation Analysis

To explore the leaders in SWC research, we analyzed the relationship between the number of articles that were published and the annual average number of cited articles in the years 1990–1995, 1996–2001, 2002–2008, 2009–2013, and 2014–2020 for nine countries.

## 3. Results

### 3.1. Characteristics of Publication Outputs

**Figure 1:**
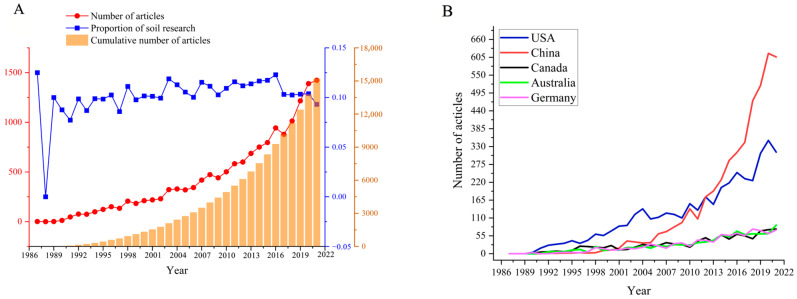
Trend of quantity that were published from 1987 to 2021 ((**A**) SWC cumulative number of articles and proportion in soil research. (**B**) Number of papers in the top five countries).

Since the 1980s, international research on SWC has increased, but the share of SWC in soil research has remained about the same. As shown in Figure 1A, the number of global SWC research papers continued to increase from 1987 to 2021, but the share of SWC in soil research fluctuated around 10% after 1990. The growth rate in the number of publications from 1987 to 2002 was relatively slow, indicating a preliminary stage of research on SWC. After 2003, the growth rate of the number of publications began to increase substantially. The five countries with the highest numbers of publications had a relatively low volume of research until 1995, with a slow growth rate in the number of publications. Then, the US emerged as the leader and had the highest number of publications until 2013, when China produced the most publications for the first time (Figure 1B). After 2013, China (3890 papers) and the US (4127 papers) became the two core forces in SWC research. 

### 3.2. Publication Categories

**Figure 2:**
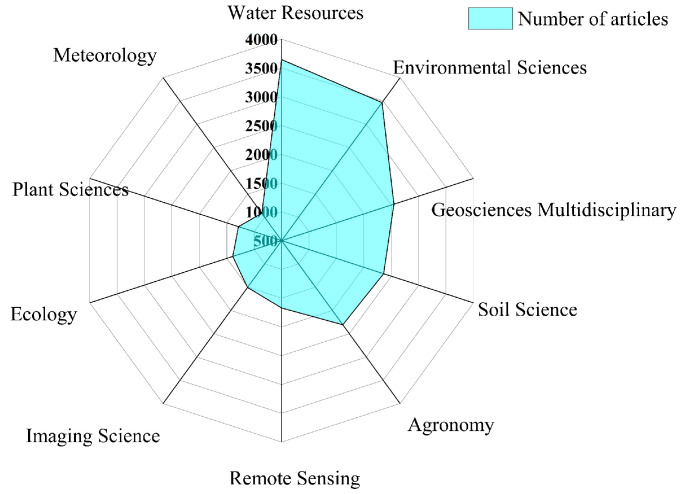
Subject categories of soil water content publications from 1987 to 2021.

Figure 2 is a radar map of the SWC research document categories. Research documents were mainly distributed in water resources, environmental sciences, geosciences multidisciplinary, soil science, agronomy, and remote sensing. Table 1 lists the primary journals related to SWC. Research on SWC thus involved a wide range of knowledge and received attention in many fields. Interdisciplinary research may be one of the important approaches to develop in the future.

### 3.3. Cooperative Network Analysis

#### 3.3.1. Cooperation among Countries

**Figure 3:**
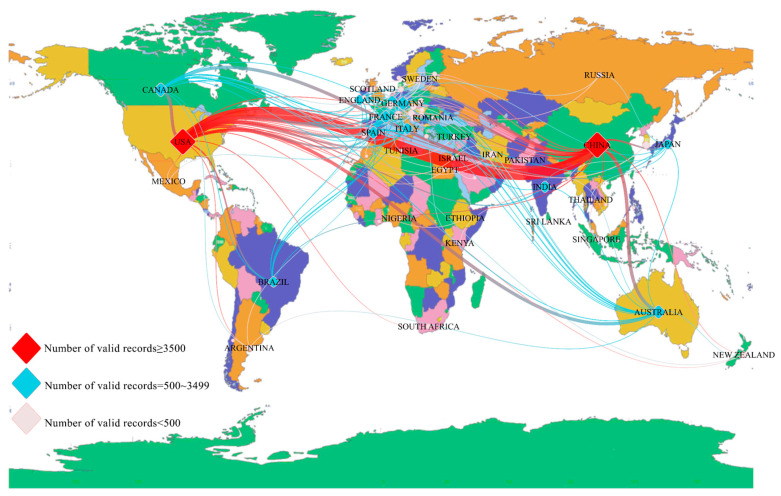
National co-occurrence map of soil water content studies from 1987 to 2021.

China and the US had the two largest nodes, with the US accumulating 4127 articles and China with slightly less, accumulating 3890 articles. Thus, China and the US occupied central positions in research on SWC. In terms of the frequency of exchanges between countries, the US had the most frequent exchanges with China, followed by Canada, Spain, Germany, Italy, Australia, and France. In addition to the US, China had frequent exchanges with Canada, Japan, and Australia but fewer with European countries, Figure 3.

The acquisition time of Table 2 was 12 August 2022. The ranking of national publications is consistent with the basic data. We think the results that were obtained can be applied to the analysis of the article. Table 2 lists the countries with more than 500 articles published, which were those countries that strongly influenced and directed scientific research on SWC. Schubert (2009) [14] introduced the single publication H-index for assessing the impact of a single publication. The US had the highest H-index (158), indicating that scientific papers in the US had the greatest influence. By contrast, India and Brazil had the lowest H-indices (<50). The Netherlands had the highest average number of citations per article (CPA), with an average of 50.66 citations per article. Although France, Italy, Spain, and England did not have particularly high numbers of articles published, the CPA of those publications was relatively high, indicating the high quality of those articles. The self-citation rate (SCR) was as high as 15.23 in China and as low as 2.44 in the UK.

#### 3.3.2. Cooperation among Institutes

**Figure 4:**
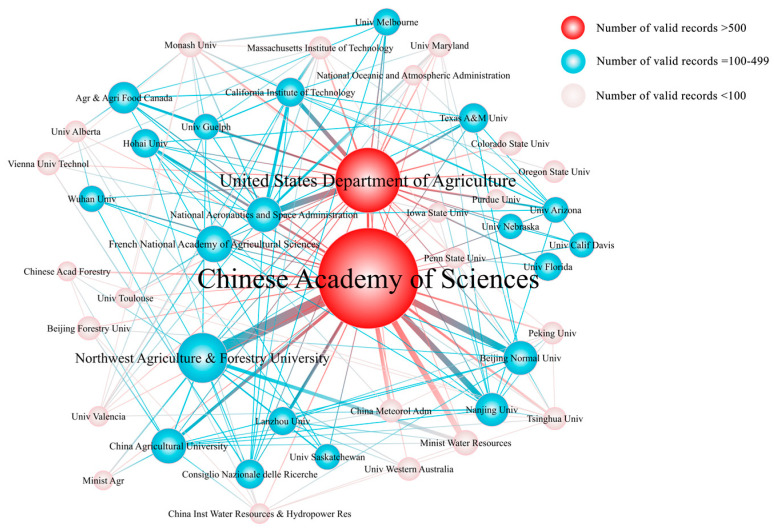
Co-occurrence map of institutions conducting research on soil water content from 1987 to 2021.

The Chinese Academy of Sciences (CAS) and the United States Department of Agriculture (USDA) occupied the top two positions in the list, reflecting great scientific capabilities in SWC research. Figure 4 shows the collaboration between SWC research institutions. Domestic institutions that had close exchanges with CAS were Northwest Agriculture and Forestry University, Beijing Forestry University, China Agricultural University, Nanjing University, Beijing Normal University, and Chinese Academy of Fishery Sciences. International exchanges were with the USDA, the French Institute of Agricultural Sciences (INRA), and Agriculture and Agri-food Canada (AAFC). Related institutions that cooperated most closely with the USDA were National Aeronautics and Space Administration (NASA), California Institute of Technology (CALTECH), Massachusetts University of Technology (MIT), and Texas A&M University. According to the results, the two national institutions stimulated research in local universities. 

### 3.4. Keywords Network Graph Characteristics

In this section, we visualized the keywords of the three regions from 1987 to 2021, and made a preliminary analysis based on the articles and keywords of the three regions in different periods.

#### 3.4.1. Research Trends in Asian and Oceanian Countries

**Figure 5:**
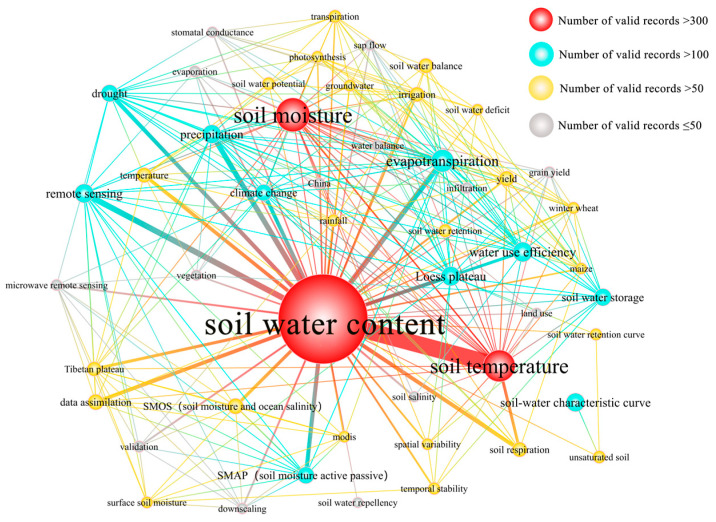
Map of keyword co-occurrence for Asia and Oceania from 1987 to 2021.

From Figure 5, we can see that SWC studies of representative countries in Asia and Oceania usually focus on the following five aspects: soil temperature, evapotranspiration, water use efficiency, Loess Plateau, and remote sensing. The soil water-heat relationship is a popular research subject in this region, but the emphasis is slightly different in different stages, as follows.

The period from 1987 to 1998 was the initial exploration stage, and research that was conducted on SWC was related to keywords such as photosynthesis, transpiration, temperature, leaf water potential, soil water potential, and hydraulic conductivity, among others. From 1999 to 2010, soil temperature, evapotranspiration, remote sensing, water use efficiency, and soil-water characteristic curve were the hot keywords. It is worth noting that the Loess Plateau also received a lot of attention. The relationship between soil hydrothermal relationships is still a research hotspot after 2010, but it is more diversified than the previous stage. In addition to the evapotranspiration, water use efficiency, and remote sensing in the previous stage, new keywords such as Loess Plateau, precipitation, drought, climate change, and data assimilation have been added, indicating that the content of SWC research continues to expand (Supplementary Figures S1–S3).

Early studies in China focused on small-scale studies such as forest [15], land, and farmland [16,17]. In the middle and late period, the research began to focus on the Loess Plateau and the relationship between land use and related land resources and SWC [18,19,20]. In addition, the research on the relationship between soil water and heat has always been one of the research hotspots in China. The early and middle studies in Japan showed little change, focusing on the influence of various influencing factors (such as drought and evapotranspiration) on the physiological ecology of vegetation [21,22,23]. In the later period, they began to pay attention to the key role of SWC in long-term regional climate change. In the early and late periods of Indian research, the impact of SWC on vegetation physiology and ecology was also studied. In the middle period, the related research of remote sensing image inversion of SWC was expanded [24,25]. Early studies in Oceania focused on soil water stress [26] and SWC transport [27], etc. In the middle stage, they mainly focused on the effects of temperature [28], evapotranspiration [29], and SWC balance [30] on plant growth [31]. In the later period, it mainly focused on the comparative verification of SMOS (soil moisture and ocean salinity) and SMAP (soil moisture active and passive) satellite SWC remote sensing products and the role of SWC in the land-air hydrological cycle.

#### 3.4.2. Research Trends in North America 

**Figure 6:**
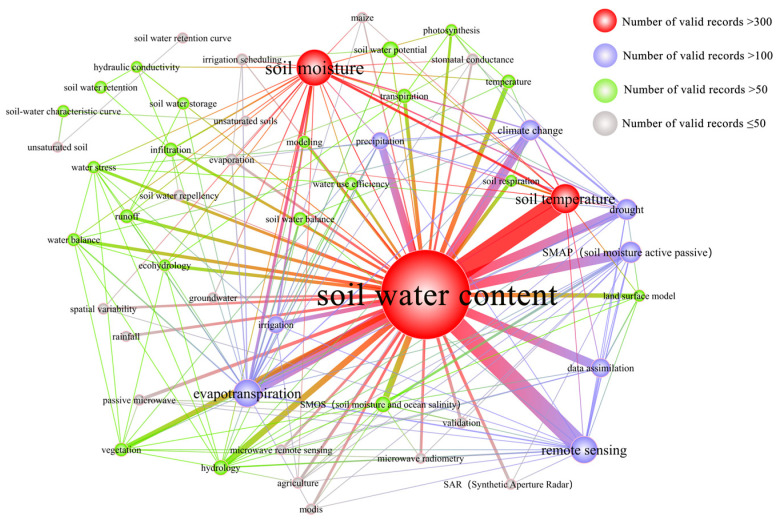
Map of keyword co-occurrence in North America from 1987 to 2021.

Figure 6 shows that SWC studies of representative countries in North America usually focus on the following five aspects: soil temperature, evapotranspiration, remote sensing, SMAP, and drought. Further analysis results are as follows. 

Between 1987 and 1998, SWC research paid increased attention to soil temperature, evapotranspiration, and soil water potential. The study of soil temperature, evapotranspiration, and remote sensing increased between 1999 and 2010. Additionally, there has been a lot of focus on hydrology, drought, climate change, soil and water potential, vegetation, and data assimilation. Soil temperature, remote sensing, and evapotranspiration were the keywords that appeared the most frequently between 2011 and 2021. Drought, climate change, and data assimilation saw a rise in frequency, and precipitation, SMAP, and SMOS satellites emerged as new hotspots (Supplementary Figures S4–S6).

In the United States, the early and middle research concentrated on the impacts of soil temperature and evapotranspiration on a few crops (such as pepper, etc.) [32], and the middle studies also broadened the pertinent studies of remote sensing and SWC [33]. Unsaturated soils and the identification and forecasting of SWC characteristic curves [34] were the main topics of early, well cited studies that were conducted in Canada. Additionally, studies on soil water potential, runoff, and stomatal conductance have been conducted, with mid-Canadian research still concentrating on how crop growth is influenced by soil water conditions. Later research in the US and Canada focused on the evaluation of SMAP satellite inversion SWC products and the establishment of related models.

#### 3.4.3. Research Trends in Europe

**Figure 7:**
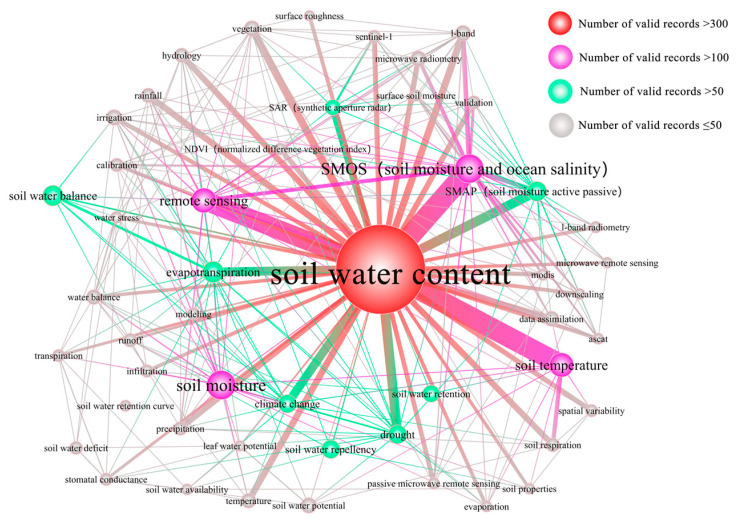
Map of keyword co-occurrence for Europe from 1987 to 1998.

Figure 7 shows that research on SWC in representative European countries generally focused on SMOS, remote sensing, and soil temperature. Different from the previous two regions which mainly studied the relationship between soil water and heat, the research of European countries took SMOS and remote sensing as the research core. The specific analysis of each period is as follows.

The terms soil water potential, SWC balance, irrigation, stress on the SWC, preferential flow, leaf water potential, soil temperature, evapotranspiration, TDR, pedotransfer functions, stomatal conductance, and other terms were used in various studies on SWC in European countries between 1987 and 1998. Evapotranspiration, remote sensing, and soil temperature were the main areas of research from 1999 to 2010. The primary research areas in European nations from 2011 to 2021 are currently SMOS inversion SWC product verification, evaluation, related field application, and data assimilation (Supplementary Figures S7–S9).

Early European studies focused on the relationship between SWC and crop physiological characteristics and vegetation growth. In addition, Germany focused on the relationship between the migration of nitrate nitrogen and SWC [35], while the UK began to pay attention to the role of SWC in climate change [36]. In the middle stage, in the context of climate change, the study of the relationship between soil CO2 emissions and SWC in tropical rain forests received attention at this stage [37]. During this period, research in the UK, France, Spain, Italy, and the Netherlands focused on the development and testing of the SMOS flight model and the development of the microwave imaging radiometer using aperture synthesis (MIRAS), thus facilitating the study of SWC microwave remote sensing inversion [38,39,40]. In the late 2011–2021 period, the research was mainly based on remote sensing products.

### 3.5. World Keyword Trend Analysis

**Figure 8:**
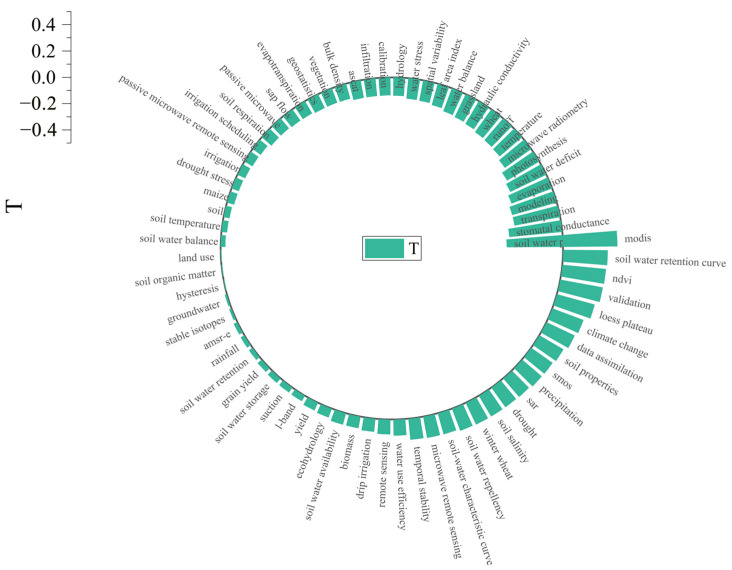
Distribution of the temporal trend of the popular keywords.

The time trend of the top 100 keywords in related research articles based on word frequency from 1987 to 2021 is shown in Figure 8. Specifically, this study used the trend factor (T) to quantify the degree of the upward or downward trend of keywords. We found that the number of studies on “ SWC potential,” “stomatal conductance,” “transpiration,” “modeling,” and “evaporation” decreased significantly in 15 years. The research on “modis”, “soil water retention Curve”, “NDVI”, and “Validation” has increased significantly after 15 years. In addition, we found that the research on “SMAP” and SWC received researchers’ attention after 15 years, and the attention was very high. It has not been in the top 100 for some time, as detailed in Supplementary Tables S1 and S2.

### 3.6. Articles Citation Influence Analysis

**Figure 9:**
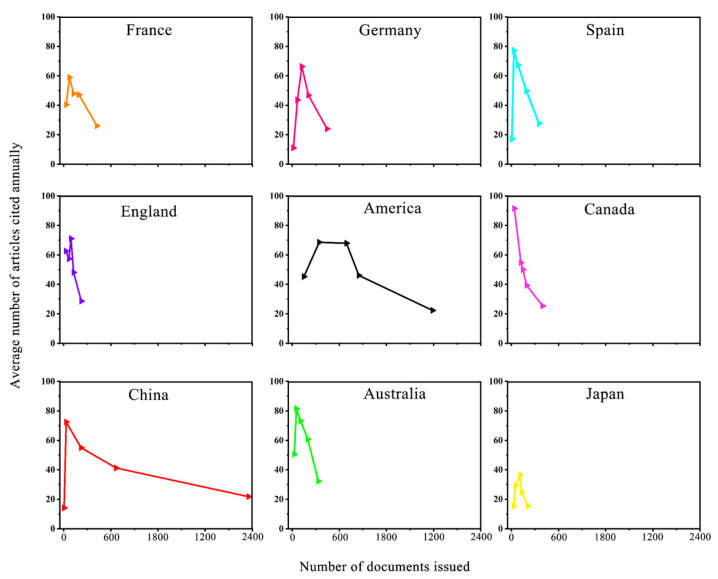
Relationships between the number of national publications and the annual citation of articles in different countries.

Each arrow in Figure 9 represents a time period, increasing from left to right. The fold-line characteristics of China and the US were similar but clearly different from those of other countries. With the continuous increase in the number of articles that were published, the average annual citation value of articles first increased and then decreased, largely because an early Canadian article was cited as many as 1904 times [41]. As a result, the fold-line of Canada was different from that of other countries, and the average annual citation value of articles showed a continuous decrease. In addition, the fold-lines of other countries were more compact because of the increase in the number of articles that were published, and the average annual citation value of articles also first increased and then decreased. However, although the number of articles that were published in China and the US continued to increase, the average number of articles cited per year did not show the same trend. Although the number of articles that were published in European countries was not large, the average number of articles that were cited per year was similar to that in the US. Therefore, although the number of articles that were published by European countries on SWC research was small, the influence of those articles should not be underestimated.

## 4. Discussion

Most of the articles in Asia from 1987 to 1998 were on the effects of SWC on plants. For example, the highly cited article “Effects of soil moisture on shrub seedling survival in a semiarid grassland” clarified how SWC affects seedling growth of grassland shrubs in semiarid areas [42]. Australia had the highest number of articles published during this period. The reliability estimation method of SWC that was proposed in “Towards areal estimation of soil water content from point measurements: time and space stability of mean responses” was cited many times between 2011 and 2021 [43]. By the period 1999 to 2010, research on SWC in Asia was dominated by agronomy, water resources, and soil science. The number of articles from China exceeded that of Australia. During this period, classic research was conducted on how SWC and soil temperature affected the growth of crops (such as corn) [44], which greatly promoted subsequent development of research. During 2011–2021, categories of articles that were issued in Asia were mainly on environmental science, water resources, and earth science. Soil water content also began to be closely associated with environmental and climate changes. Therefore, research in this period was interdisciplinary and digitalized. Remote sensing technology was introduced and emerged in SWC research to begin a new research direction.

Soil water content-related research in North America from 1987 to 1998 was also mostly related to agriculture, with soil science, plant science, and agronomy being the primary three disciplines. The classic article “Equations for the soil-water characteristic curve” published by Canadian scholars during this period was cited 1904 times in the past 10 years, laying the foundation for subsequent soil physics research [41]. Categories of articles that were published from 1999 to 2010 mainly included water resources, environmental science, and soil science. The most striking factor was that remote sensing technology began to be used in research on SWC. “The Soil Moisture Active Passive (SMAP) Mission” [45] was cited frequently at 1939 times, demonstrating its tremendous influence. By the 2011 to 2021 stage, SWC research, similar to Asian countries, was largely linked to environment and climate change and was developed by NASA (National Aeronautics and Space Administration). The SMAP satellites that began to provide global SWC data in 2015 contributed to improving agricultural drought monitoring and forecasting. 

Research on SWC in European countries from 1987 to 1998 focused on soil science, agronomy, and water resources. The representative article “Effects of thinning on soil and tree water relations, transpiration and growth in an oak forest (Quercus petraea (Matt) Liebl)” quantified relationships among SWC, stomatal conductance, and growth of the stand [46], and it greatly affected subsequent research. From 1999 to 2010, remote sensing technology reached relative maturity in European SWC research. The SMOS satellite that was launched by the European Space Agency in 2009 was a milestone remote sensing project to acquire SWC. The most cited pioneering article “The SMOS mission: new tools for monitoring key elements of the global water cycle” expanded on how SMOS observes global SWC [47] and had wide and far-reaching influence. In the 2011 to 2021 period, environmental science, water resources, and remote sensing were the top three categories of documents. Remote sensing technology, SMOS, and SMAP became important branches of SWC research in European countries. SMAP has gained a lot of attention worldwide from 2015 to 2021.

Future study directions are anticipated based on the literature review of SWC presented above as follows:

(1) The development of remote sensing technology brings new opportunities to SWC research, but also brings the problem of uncertainty of monitoring data. How to obtain products with high precision, continuous time, and low price through technological upgrading will greatly promote relevant research.
(2) More attention should be paid to the tradeoff between soil moisture and ecological engineering in ecologically fragile regions, such as the Loess Plateau and karst region in China.
(3) Soil moisture is closely related to agricultural production. In the context of tight global food security, how to ensure the efficient use of farmland SWC is a major issue of water resources in arid and semi-arid regions.

## 5. Conclusions

Since 1987, the number of international SWC research documents increased rapidly, especially the number of articles that were published from 2011 to 2021, which accounted for 68% of the total number of articles that were published. The results of the bibliometric analysis showed that China and the US had the largest number of articles published on SWC research and that they also cooperated closely. However, compared with the US, China had fewer exchanges with European countries. The CAS and USDA were the two institutions with the largest number of articles published, and they were closely linked with national agricultural and forestry research institutions, showing strong scientific research capabilities and influence. The SWC research papers were mainly distributed in the categories of water resources, environmental sciences, geosciences multidisciplinary, soil science, agronomy, and remote sensing.

To reveal the histories and hotspots of research on SWC in different regions of the world in different periods, this study analyzed research trends in SWC in the three major regions of Asia and Oceania, North America, and Europe during the periods 1987 to 1998, 1999 to 2010, and 2011 to 2021. The main conclusions were as follows:

1. From 1987 to 1998, global SWC research focused on SWC stress, soil hydrothermal processes, soil physics, and SWC measurement methods, among other areas, and research objects were mostly small-scale units such as farmlands and woodlands. Research in China was in the initial learning stage. Research focused on agricultural and animal husbandry production, and there were many cases of international exchanges and cooperation. Japan and Australia have focused on mechanistic research and have started using models as a result of accumulating long-term monitoring data, whereas North American countries began to focus on the effects of SWC on surface evapotranspiration. Research directions in Europe at this stage became diversified and decentralized, and there were many pioneering studies.
2. From 1999 to 2010, the major change was that China was affected by policies and showed an explosive increase in SWC research. Research directions were diverse, and research objects were biased toward basin-scale SWC monitoring and mechanistic research. Europe focused on satellite research and development and testing at this stage, which promoted the process of remote sensing inversion of SWC. Individual countries closely analyzed relationships between SWC and greenhouse gas emissions under the background of climate change. Research directions in North America were diverse, and the research results showed an explosive increase, with evapotranspiration remaining a research hotspot and SWC products obtained from satellite inversion beginning to be applied.
3. From 2001 to 2021, SWC research in China remained concentrated on the Loess Plateau, and diversity of research directions increased. Development of remote sensing technology promoted the successful acquisition of soil products at regional scales, contributing to the assessment of global climate change together with other parameters. In North America, SWC research focused on the evaluation and improvement of satellite inversion SWC products. As an important factor, SWC participates in land–air energy exchange. In Europe, SWC research at this stage focused on SMAP and SMOS and continued to deepen understanding of remote sensing SWC inversion. Although the number of articles that were published was small, those articles were highly influential.
4. Compared with traditional methods to measure SWC, remote sensing had considerable advantages in large-scale monitoring of SWC and was also a research hotspot on global SWC at this stage. However, to obtain SWC products with high accuracy and high temporal and spatial consistency based on long-term field observations, it remained important to clarify responses and feedback mechanisms of SWC in processes of land surface water and gas circulation.

## Figures and Tables

**Table 1:** Top 15 journals by volume of publications on soil water content.

Item	Journal	Impact Factors of 2021
1	Journal of Hydrology	6.708
2	Agricultural Water Management	6.611
3	Remote Sensing	5.349
4	IEEE Transactions on Geoscience and Remote Sensing	8.125
5	Hydrological Processes	3.784
6	Remote Sensing of Environment	13.85
7	Geoderma	7.422
8	Plant and Soil	4.993
9	Water	3.53
10	Soil Tillage Research	7.366
11	Agricultural and Forest Meteorology	6.424
12	Catena	6.367
13	Science of the Total Environment	10.753
14	IEEE Journal of Selected Topics in applied Earth Observations and Remote Sensing	4.715
15	Water Resources Research	6.159

**Table 2:** Countries with high publication productivity.

Country	TA	TC	CPA	SCR	H-Index
USA	4128	89981	36.36	7.70	158
China	3897	43830	19.17	15.23	99
Canada	975	24055	32.37	3.90	75
Australia	937	23237	33.02	3.69	82
Germany	931	24098	32.73	3.44	83
France	785	21149	44.46	7.13	88
Spain	731	18389	37.96	5.39	79
India	680	9151	15.34	4.24	46
Italy	659	18404	43.1	5.20	78
England	605	19914	40.43	2.44	76
Brazil	580	8170	17.03	4.82	47
Japan	572	10293	20.66	3.06	53
Netherlands	505	17487	50.66	4.08	80

TA: total articles; TC: total citations; CPA: average number of citations per article; SCR: self-citation rate (%); H-index: H papers that have been cited at least H times.

## Data Availability

The datasets used or analyzed during the current study are available from the corresponding author on reasonable request.

## Supplementary Materials

The following supporting information can be downloaded at: https://www.mdpi.com/article/10.3390/ijerph192315476/s1, Figure S1: Map of keywords co-occurrence for Asia and Oceania from 1987 to 1998;Figure S2: Map of keyword co-occurrence for Asia and Oceania from 1999 to 2010; Figure S3: Map of keyword co-occurrence for Asia and Oceania from 2011 to 2021; Figure S4: Map of keyword co-occurrence in North America from 1987 to 1998; Figure S5: Map of keyword co-occurrence in North America from 1999 to 2010; Figure S6: Map of keyword co-occurrence in North America 2011 to 2021; Figure S7: Map of keyword co-occurrence for Europe from 1987 to 1998; Figure S8: Map of keyword co-occurrence for Europe from 1999 to 2010; Figure S9: Map of keyword co-occurrence for Europe from 2011 to 2021; Table S1: Normalized keyword frequency FR and trend factor T from 2015 to 2021. Table S2: Normalized keyword frequency FP from 1987 to 2014.

## References

[1] Mason P.J., Zillman J.W., Simmons A., Lindstrom E.J., Harrison D.E., Dolman H., Bojinski S., Fischer A., Latham J., Rasmussen J. (2010). Implementation Plan for the Global Observing System for Climate in Support of the UNFCCC (2010 Update). Lect. Notes Phys..

[2] Raats P.A.C. (2001). Developments in soil-water physics since the mid 1960s. Geoderma.

[3] Richards L.A. (1931). Capillary conduction of liquids through porous mediums. Phys.-A J. Gen. Appl. Phys..

[4] Daly E., Porporato A. (2005). A review of soil moisture dynamics: From rainfall infiltration to ecosystem response. Environ. Eng. Sci..

[5] Seneviratne S.I., Corti T., Davin E.L., Hirschi M., Jaeger E.B., Lehner I., Orlowsky B., Teuling A.J. (2010). Investigating soil moisture-climate interactions in a changing climate: A review. Earth-Sci. Rev..

[6] Dobriyal P., Qureshi A., Badola R., Hussain S.A. (2012). A review of the methods available for estimating soil moisture and its implications for water resource management. J. Hydrol..

[7] Famiglietti J.S., Ryu D., Berg A.A., Rodell M., Jackson T.J. (2008). Field observations of soil moisture variability across scales. Water Resour. Res..

[8] Peng J., Albergel C., Balenzano A., Brocca L., Cartus O., Cosh M.H., Crow W.T., Dabrowska-Zielinska K., Dadson S., Davidson M.W.J. (2021). A roadmap for high-resolution satellite soil moisture applications—Confronting product characteristics with user requirements. Remote Sens. Environ..

[9] Brooks S.M., Richards K.S. (1994). The significance of rainstorm variations to shallow translational hillslope failure. Earth Surf. Process. Landf..

[10] Andren O., Rajkai K., Katterer T. (1993). Water and temperature dynamics in a clay soil under winter-wheat—Influence on straw decomposition and n-immobilization. Biol. Fertil. Soils.

[11] Yoshioka M., Takakura S., Ishizawa T., Sakai N. (2015). Temporal changes of soil temperature with soil water content in an embankment slope during controlled artificial rainfall experiments. J. Appl. Geophys..

[12] Wang X., Zhang S., Liu Y. (2021). ITGInsight–discovering and visualizing research fronts in the scientific literature. Scientometrics.

[13] Zhu J.-J., Dressel W., Pacion K., Ren Z.J. (2021). ES&T in the 21st Century: A Data-Driven Analysis of Research Topics, Interconnections, And Trends in the Past 20 Years. Environ. Sci. Technol..

[14] Schubert A. (2009). Using the h-index for assessing single publications. Scientometrics.

[15] Wang Y.H. (1992). The hydrological influence of black locust plantations in the loess area of Northwest China. Hydrol. Process..

[16] Li J.S., Kawano H. (1996). The areal distribution of soil moisture under sprinkler irrigation. Agric. Water Manag..

[17] Zhu Z.X., Stewart B.A., Fu X.J. (1994). Double cropping wheat and corn in a subhumid region of China. Field Crops. Res..

[18] Huang Y., Chen L., Fu B., Huang Z., Gong J. (2005). The wheat yields and water-use efficiency in the Loess Plateau: Straw mulch and irrigation effects. Agric. Water Manag..

[19] Qiu Y., Fu B.J., Wang J., Chen L.D. (2001). Soil moisture variation in relation to topography and land use in a hillslope catchment of the Loess Plateau, China. J. Hydrol..

[20] Qiu Y., Fu B., Wang J., Chen L. (2003). Spatiotemporal prediction of soil moisture content using multiple-linear regression in a small catchment of the Loess Plateau, China. Catena.

[21] Lee C.Y., Tsuno Y., Nakano J., Yamaguchi T. (1994). Ecophysiological Studies on the Drought Resistance of Soybean.1. Changes in Photosynthesis, Transpiration and Root Respiration with Soil-Moisture Deficit. Jpn. J. Crop. Sci..

[22] Ohte N., Koba K., Yoshikawa K., Sugimoto A., Matsuo N., Kabeya N., Wang L. (2003). Water utilization of natural and planted trees in the semiarid desert of Inner Mongolia, China. Ecol. Appl..

[23] Yanagisawa N., Fujita N. (1999). Different distribution patterns of woody species on a slope in relation to vertical root distribution and dynamics of soil moisture profiles. Ecol. Res..

[24] Dyi-Huey C., Kothari R., Islam S. (2003). Classification of soil texture using remotely sensed brightness temperature over the Southern Great Plains. IEEE Trans. Geosci. Remote Sens..

[25] Singh D. (1999). Effect of soil moisture on microwave scattering for remote sensing. Sadhana-Acad. Proc. Eng. Sci..

[26] Sasse J., Sands R. (1996). Comparative responses of cuttings and seedlings of Eucalyptus globulus to water stress. Tree Physiol..

[27] Western A.W., Bloschl G., Grayson R.B. (1998). How well do indicator variograms capture the spatial connectivity of soil moisture?. Hydrol. Process..

[28] Hoyle G.L., Steadman K.J., Daws M.I., Adkins S.W. (2008). Pre- and post-harvest influences on seed dormancy status of an Australian Goodeniaceae species, Goodenia fascicularis. Ann. Bot..

[29] Payero J.O., Tarkalson D.D., Irmak S., Davison D., Petersen J.L. (2009). Effect of timing of a deficit-irrigation allocation on corn evapotranspiration, yield, water use efficiency and dry mass. Agric. Water Manag..

[30] Melland A.R., Vigiak O., Roberts A.M., Rattray D., Whitford J. (2010). Evaluation of a static water balance model in cropped and grazed systems of temperate Australia. Environ. Model. Softw..

[31] Latta R.A., Cocks P.S., Matthews C. (2002). Lucerne pastures to sustain agricultural production in southwestern Australia. Agric. Water Manag..

[32] Smittle D.A., Dickens W.L., Stansell J.R. (1994). Irrigation regimes affect yield and water-use by bell pepper. J. Am. Soc. Hortic. Sci..

[33] Jacobs J.M., Myers D.A., Whitfield B.M. (2003). Improved rainfall/runoff estimates using remotely sensed soil moisture. J. Am. Water Resour. Assoc..

[34] Fredlund D.G., Xing A.Q. (1994). Equations for the soil-water characteristic curve. Can. Geotech. J..

[35] Bredemeier M., Blanck K., Lamersdorf N., Wiedey G.A. (1995). Response of soil-water chemistry to experimental clean rain in the nitrex roof experiment at selling, Germany. For. Ecol. Manag..

[36] Rowntree P.R., Murphy J.M., Mitchell J.F.B. (1993). Climatic-change and future rainfall predictions. J. Inst. Water Environ. Manag..

[37] Sotta E.D., Veldkamp E., Guimarães B.R., Paixão R.K., Ruivo M.L.P., Almeida S.S. (2006). Landscape and climatic controls on spatial and temporal variation in soil CO2 efflux in an Eastern Amazonian Rainforest, Caxiuanã, Brazil. For. Ecol. Manag..

[38] Colliander A., Jackson T.J., Bindlish R., Chan S., Das N., Kim S.B., Cosh M.H., Dunbar R.S., Dang L., Pashaian L. (2017). Validation of SMAP surface soil moisture products with core validation sites. Remote Sens. Environ..

[39] Martin-Neira M., Cabeza I., Perez C., Palacios M.A., Guijarro M.A., Ribo S., Corbella I., Blanch S., Torres F., Duffo N. (2008). AMIRAS—An airborne MIRAS demonstrator. IEEE Trans. Geosci. Remote Sens..

[40] Mecklenburg S., Drusch M., Kaleschke L., Rodriguez-Fernandez N., Reul N., Kerr Y., Font J., Martin-Neira M., Oliva R., Daganzo-Eusebio E. (2016). ESA’s Soil Moisture and Ocean Salinity mission: From science to operational applications. Remote Sens. Environ..

[41] Fredlund D.G., Xing A.Q., Huang S.Y. (1994). Predicting the permeability function for unsaturated soils using the soil-water characteristic curve. Can. Geotech. J..

[42] Harrington G.N. (1991). Effects of soil-moisture on shrub seedling survival in a semiarid grassland. Ecology.

[43] Grayson R.B., Western A.W. (1998). Towards areal estimation of soil water content from point measurements: Time and space stability of mean response. J. Hydrol..

[44] Zhou L.-M., Li F.-M., Jin S.-L., Song Y. (2009). How two ridges and the furrow mulched with plastic film affect soil water, soil temperature and yield of maize on the semiarid Loess Plateau of China. Field Crops. Res..

[45] Entekhabi D., Njoku E.G., O’Neill P.E., Kellogg K.H., Crow W.T., Edelstein W.N., Entin J.K., Goodman S.D., Jackson T.J., Johnson J. (2010). The Soil Moisture Active Passive (SMAP) Mission. Proc. IEEE.

[46] Breda N., Granier A., Aussenac G. (1995). Effects of thinning on soil and tree water relations, transpiration and growth in an oak forest (*Quercus petraea* (Matt.) Liebl.). Tree Physiol.

[47] Kerr Y.H., Waldteufel P., Wigneron J.-P., Delwart S., Cabot F., Boutin J., Escorihuela M.-J., Font J., Reul N., Gruhier C. (2010). The SMOS Mission: New Tool for Monitoring Key Elements ofthe Global Water Cycle. Proc. IEEE.

