# Supplementary material for: Evolution of Research on Global Soil Water Content in the Past 30 Years Based on ITGinsight Bibliometric Analysis
Source: Int J Environ Res Public Health. 2022 Nov 22;19(23):15476. doi: 10.3390/ijerph192315476 (PMC9740670; doi:10.3390/ijerph192315476)
Supplement: Supplementary file 1 [file ijerph-19-15476-s001.zip › supplement figures.pdf]

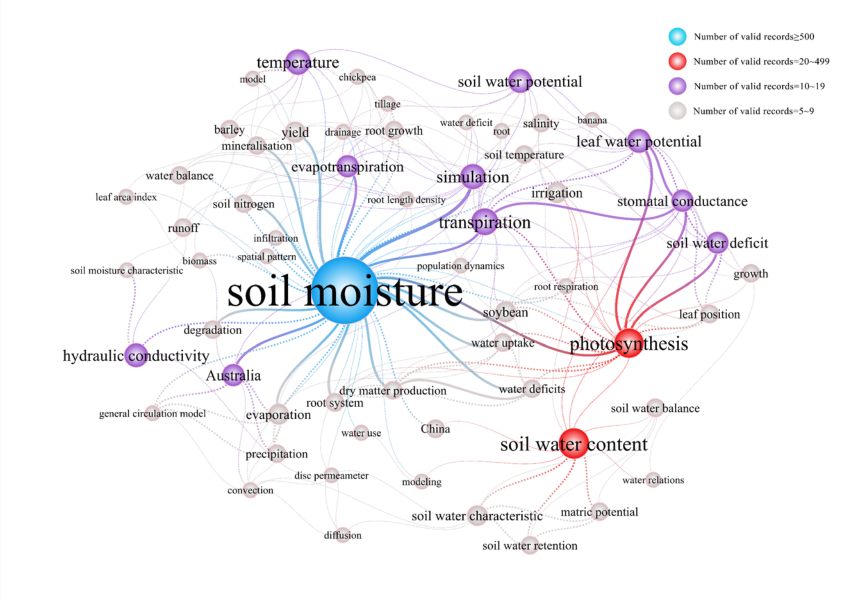

Figure S1. Map of keywords co-occurrence for Asia and Oceania from 1987 to 1998

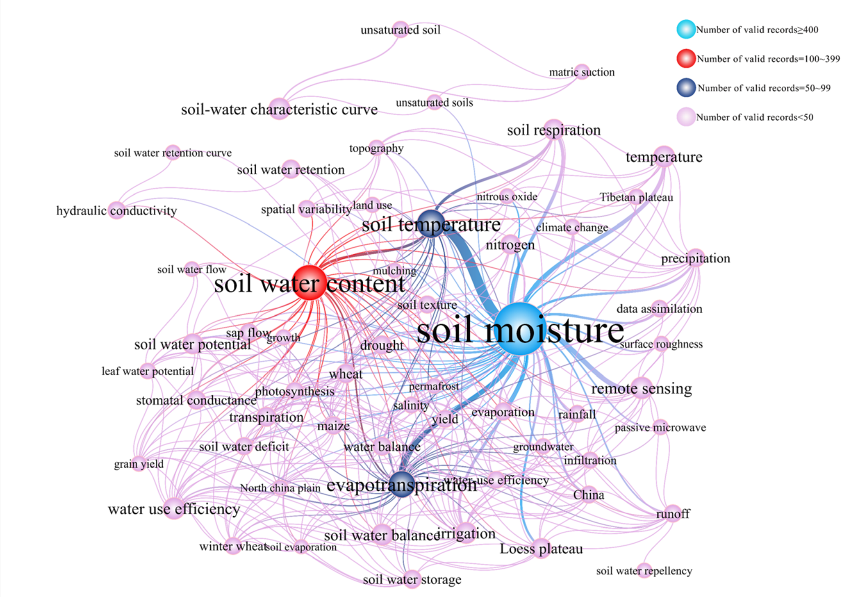

Figure S2. Map of keyword co-occurrence for Asia and Oceania from 1999 to 2010

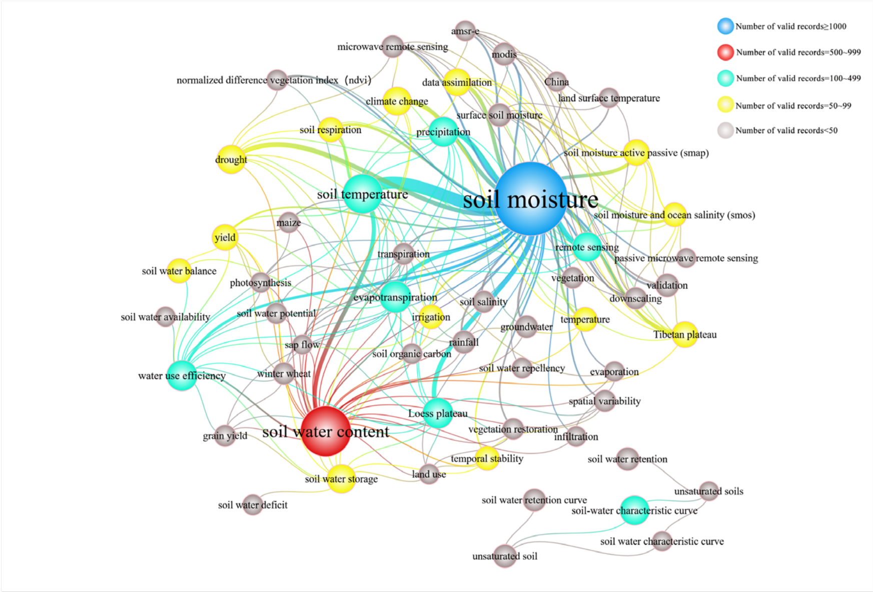

Figure S3. Map of keyword co-occurrence for Asia and Oceania from 2011 to 2021

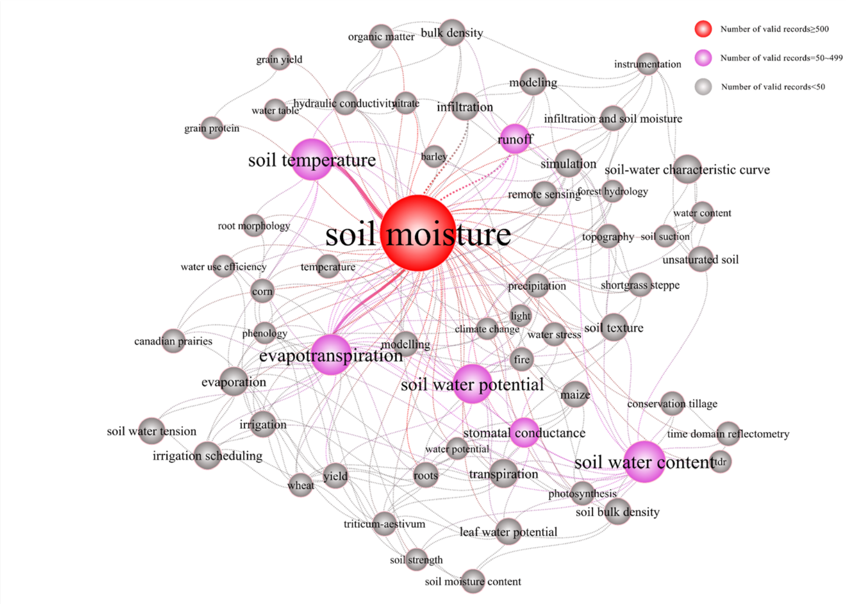

Figure S4. Map of keyword co-occurrence in North America from 1987 to 1998

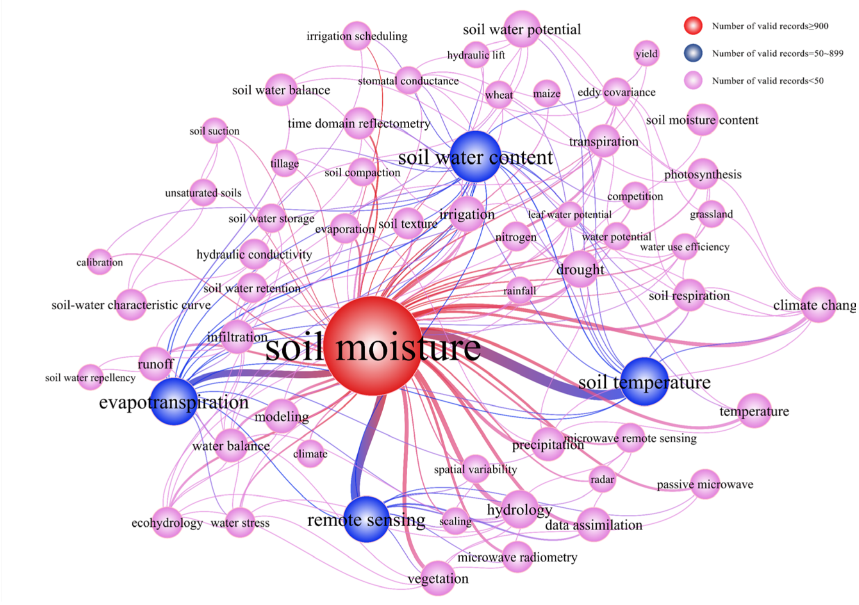

Figure S5. Map of keyword co-occurrence in North America from 1999 to 2010

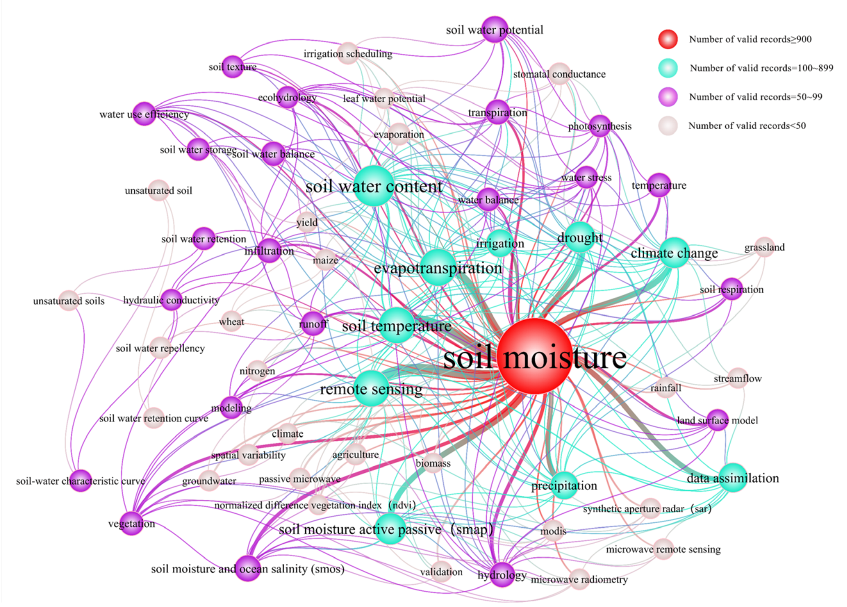

Figure S6. Map of keyword co-occurrence in North America 2011 to 2021

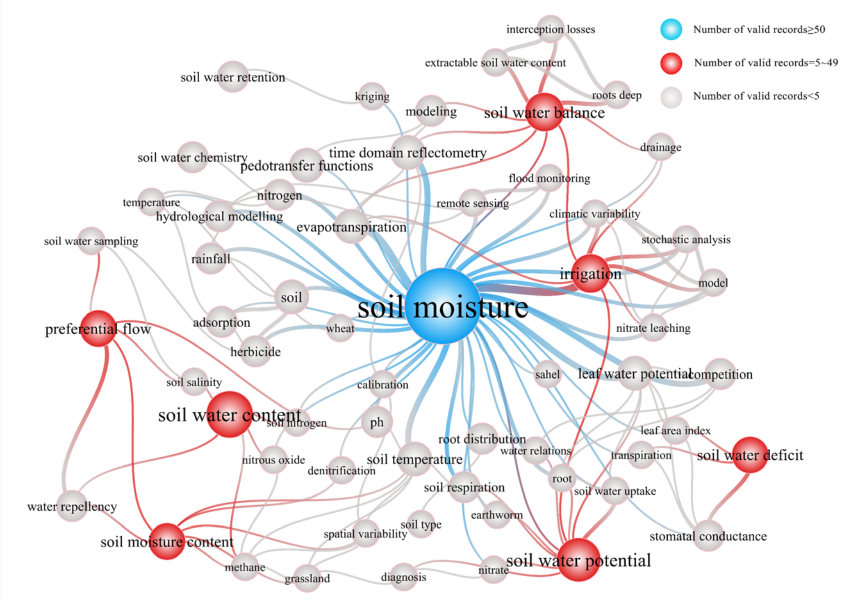

Figure S7. Map of keyword co-occurrence for Europe from 1987 to 1998

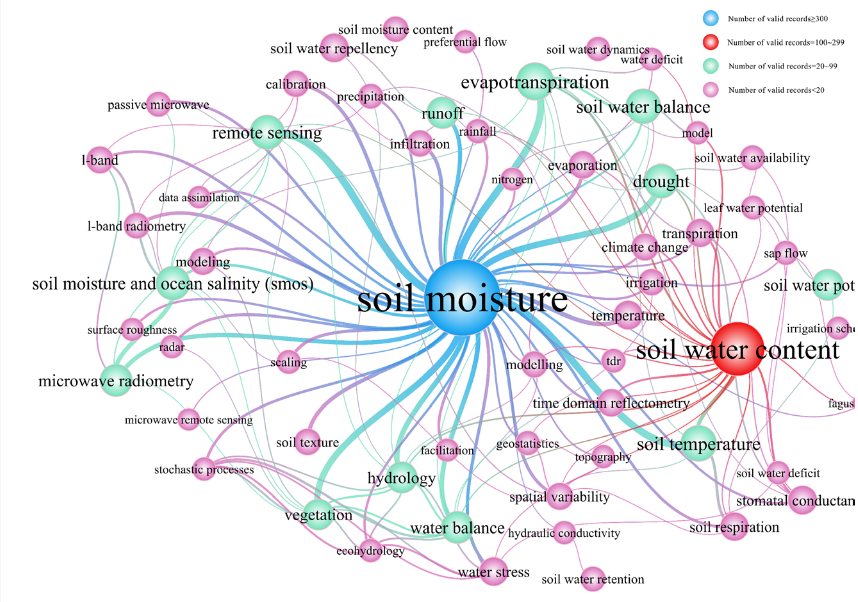

Figure S8. Map of keyword co-occurrence for Europe from 1999 to 2010

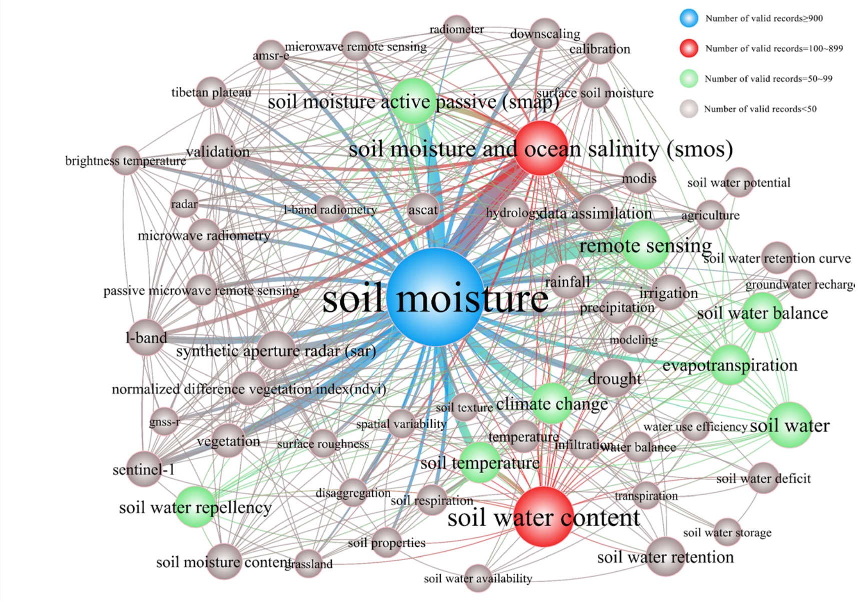

Figure S9. Map of keyword co-occurrence for Europe from 2011 to 2021
