# Supplementary material for: Evolution of Research on Global Soil Water Content in the Past 30 Years Based on ITGinsight Bibliometric Analysis
Source: Int J Environ Res Public Health. 2022 Nov 22;19(23):15476. doi: 10.3390/ijerph192315476 (PMC9740670; doi:10.3390/ijerph192315476)
Supplement: Supplementary file 1 [file ijerph-19-15476-s001.zip › supplement tables.pdf]

Table S1. Normalized keyword frequency FR and trend factor T from 2015 to 2021

keywords	F_R	T	keywords	F_R	T
soil temperature	53.26	-0.04	spatial variability	7.180157	-0.16
remote sensing	38.90	0.11	soil water deficit	6.92	-0.30
evapotranspiration	34.60	-0.11	bulk density	6.66	-0.12
smap	31.07	#	water stress	6.66	-0.16
climate change	29.77	0.25	ascat	6.40	-0.14
drought	28.33	0.18	soil moisture sensor	6.40	#
smos	24.15	0.20	winter wheat	6.27	0.18
precipitation	22.98	0.20	soil salinity	6.27	0.18
soil-water characteristic curve	20.23	0.17	irrigation scheduling	6.01	-0.08
data assimilation	19.06	0.24	synthetic aperture radar	5.87	#
soil water balance	18.80	-0.04	biomass	5.87	0.09
soil water retention	17.36	0.03	calibration	5.61	-0.14
water use efficiency	17.23	0.12	land surface temperature	5.61	#
soil water retention curve	17.10	0.34	ecohydrology	5.48	0.07
irrigation	16.97	-0.07	crop yield	5.48	#
soil water repellency	15.67	0.18	l-band	5.48	0.05
soil water storage	13.97	0.04	drip irrigation	5.35	0.10
loess plateau	12.14	0.30	agricultural drought	5.35	#
yield	12.01	0.06	land use	5.35	-0.01
temperature	11.49	-0.22	atmosphere-land interaction	5.22	#
soil respiration	11.23	-0.10	monitoring	5.22	#
modis	10.97	0.41	deficit irrigation	5.221932	#
rainfall	10.44	0.03	sap flow	5.091384	-0.11
infiltration	9.40	-0.14	photosynthesis	5.091384	-0.29
water balance	9.14	-0.19	evaporation	4.960836	-0.33
ndvi	9.14	0.33	grain yield	4.960836	0.04
surface soil moisture	8.75	#	stomatal conductance	4.960836	-0.41
sar	8.75	0.19	groundwater recharge	4.960836	#
soil water potential	8.75	-0.43	microwave radiometry	4.960836	-0.25
downscaling	8.49	#	uncertainty	4.830287	#
runoff	8.49	-0.22	grassland	4.830287	-0.19
validation	8.49	0.33	hydrus-1d	4.699739	#
sentinel-1	8.36	#	random forest	4.699739	#
vegetation	8.09	-0.12	wheat	4.699739	-0.21
agriculture	7.83	#	amsr2	4.699739	#
land surface model	7.83	#	amsr-e	4.699739	0.03
hydraulic conductivity	7.83	-0.20	soil organic matter	4.569191	-0.01
soil water availability	7.83	0.09	suction	4.569191	#
modeling	7.83	-0.35	passive microwave	4.438642	-0.10
microwave remote sensing	7.57	0.17	stable isotopes	4.438642	0.02
soil properties	7.57	0.21	vapor pressure deficit	4.438642	#
machine learning	7.57	#	soil measurements	4.308094	#
biochar	7.57	#	passive microwave remote sensing	4.046997	-0.07
groundwater	7.57	0.02	leaf area index	4.046997	-0.17
soil organic carbon	7.44	#	drought stress	4.046997	-0.06
tibetan plateau	7.44	#	swat	4.046997	#

hydrology	7.31	-0.14	soil	4.046997	-0.05
maize	7.31	-0.06	triple collocation	4.046997	#
temporal stability	7.31	0.16	geostatistics	4.046997	-0.12
transpiration	7.18	-0.35	hysteresis	3.916449	-0.01

# : indicates that the keyword entered the top 100 only after 2015

Table S2. Normalized keyword frequency FP from 1987 to 2014

keywords	FP	note	keywords	FP	note
soil temperature	58.93		sap flow	6.50	
evapotranspiration	44.46		salinity	6.50	*
remote sensing	29.99		soil water availability	6.37	
soil water potential	23.49		topography	6.24	*
soil water balance	20.44		loess plateau	6.10	
irrigation	19.77		leaf area index	5.97	
temperature	19.24		sar	5.71	
drought	18.58		competition	5.71	*
modeling	17.65		water potential	5.71	*
climate change	16.59		simulation	5.71	*
transpiration	16.19		passive microwave	5.57	
soil water retention	16.06		land use	5.44	
smos	15.26		model	5.44	*
precipitation	14.60		growth	5.44	*
soil respiration	14.07		soil bulk density	5.31	*
water balance	14.07		geostatistics	5.31	
runoff	14.07		water-use efficiency	5.18	*
soil water deficit	13.93		microwave remote sensing	5.18	
soil-water characteristic curve	13.80		climate	5.04	*
water use efficiency	13.01		soil water dynamics	5.04	*
infiltration	12.87		temporal stability	5.04	
soil water storage	12.61		soil structure	4.91	*
stomatal conductance	12.61		l-band	4.91	
hydraulic conductivity	12.48		nitrous oxide	4.78	*
nitrogen	11.55	*	eddy covariance	4.78	*
data assimilation	11.02		passive microwave remote sensing	4.78	
vegetation	10.62		biomass	4.78	
evaporation	10.62		preferential flow	4.64	*
yield	10.35		ecohydrology	4.64	
soil water repellency	10.35		soil organic matter	4.64	
spatial variability	10.35		drought stress	4.64	
hydrology	10.09		soil properties	4.64	
photosynthesis	9.82		grain yield	4.51	
leaf water potential	9.82	*	soil	4.51	
rainfall	9.69		amsr-e	4.38	
water stress	9.56		drip irrigation	4.25	
bulk density	8.76		stable isotopes	4.25	
microwave radiometry	8.76		ndvi	4.25	
time domain reflectometry	8.76	*	modis	4.25	
maize	8.36		nitrate	4.11	*
soil water retention curve	7.83		soil salinity	4.11	
calibration	7.70		matric suction	4.11	
wheat	7.70		winter wheat	4.11	
grassland	7.56		soil moisture dynamics	3.98	*
tdr	7.43	*	forest	3.98	*

groundwater	7.30		soil nitrogen	3.98	*
irrigation scheduling	7.17		validation	3.98	
drainage	6.77	*	hysteresis	3.98	
soil compaction	6.64	*	root distribution	3.85	*
tillage	6.64	*	pedotransfer functions	3.85	*

* : Keywords not listed in the 100 list after 2015
